# LncRNA DLEU1 Contributes to the Growth and Invasion of Colorectal Cancer *via* Targeting miR-320b/PRPS1

**DOI:** 10.3389/fonc.2021.640276

**Published:** 2021-05-25

**Authors:** Dong Xu, Fei Yang, Yongchao Fan, Wanling Jing, Jianfei Wen, Wen Miao, Xiaoyan Ding, Hongbao Yang

**Affiliations:** ^1^ Department of General Surgery, Gaochun People’s Hospital, Nanjing, China; ^2^ Department of Internal Medicine, Gaochun People’s Hospital, Nanjing, China; ^3^ Center for New Drug Safety Evaluation and Research, Institute of Pharmaceutical Science, China Pharmaceutical University, Nanjing, China; ^4^ Department of General Surgery, The First Affiliated Hospital of Nanjing Medical University, Nanjing, China

**Keywords:** colorectal cancer, lncRNA, DLEU1, miR-320b, PRPS1

## Abstract

Growing evidences suggest that long non-coding RNAs (lncRNAs) are closely correlated to the development of human cancer, such as colorectal cancer (CRC). A previous report suggested that DLEU1 accelerated CRC development. However, DLEU1’s underlying mechanism in CRC remains unclear. In our study, the level of DLEU1 in CRC tissues is investigated by qRT-PCR. Our data exhibited that DLEU1 level was observably increased in CRC tissues and CRC cell lines and was closely associated with bad prognosis of CRC patients. CRC cell proliferation was repressed by sh-LncRNA DLEU1, whereas cell apoptosis was markedly stimulated. Moreover, knockdown of DLEU1 inhibited cell migration and invasion. Mechanistically, through interacting with miR-320b in CRC, DLEU1 promoted the level of PRPS1 which was a target of miR-320b. The rescue experiment confirmed that knockdown of DLEU1 repressed cell proliferation, migration and invasion while stimulated cell apoptosis via miR-320b/phosphoribosyl pyrophosphate synthetase 1 (PRPS1) axis. Meanwhile, the data of xenograft model exhibited that inhibition of DLEU1 suppressed tumor growth *in vivo*. In summary, DLEU1 knockdown may repress PRPS1 expression via miR-320b, and then repress cell proliferation, migration and invasion while stimulate cell apoptosis. Our research may provide a novel target for the treatment of CRC.

## Introduction

Colorectal cancer (CRC) is the third most common malignant tumor globally (1). In China, CRC has the fourth highest malignancies incidence and accounts for 9.2% of the total cancer death (2). Genetic variation and cellular environment are the main causes affecting tumor occurrence, development and metastasis (3). Despite the development of surgery, chemotherapy, radiotherapy and immunotherapy, the recurrence and metastasis rate is still high in 70% of patients with stage 2 and 3 of CRC (4). However, the understanding of molecular mechanisms of proliferation, apoptosis, migration and invasion is extremely limited. A deep understanding of the invasion and development of CRC is critical to accelerate the development of its diagnosis and treatment.

Emerging evidences suggest that long non-coding RNAs (lncRNAs) are closely related to the development of human cancer and regulate several cell functions (5). A lot of lncRNAs have been found to inhibit or promote the development of CRC. For evidence, lncRNA ZNF667-AS1 and lncRNA XIST can suppress the invasion, development and migration of CRC (6, 7). Nevertheless, lncRNA SNHG16, LINC00460 and IncARSR may act essential roles in promoting the development of CRC (8–10).

The lncRNA deleted in lymphocytic leukemia 1 (DLEU1) located on chromosome 13q14.3 has been reported to be abnormally expressed in many cancers (11). DLEU1 is obviously overexpressed in breast cancer and accelerates migration, invasion of breast cancer cells (12). Besides, DLEU1 is also found to be highly expressed in osteosarcoma (13), glioblastoma multiforme (14) and hepatocellular carcinoma (15), and aggravates cancer progression.

Recent studies reported that lncRNAs play an important role in tumor occurrence and development by sponging miRNA to modulate the expression of protein-coding genes (16). DLEU1 accelerates cell proliferation and invasion in bladder cancer via miR-99b/HS3ST3B1 Axis (17). A study conducted by Liu et al. (18) found that through sponging miR-381 and elevating HOXA13 level in cervical cancer, DLEU1 stimulates cell proliferation and invasion. However, the significant role of DLEU1 in the development of CRC remains unclear.

In this study, we investigated the effect of DLEU1 on the development of CRC and established a competitive endogenous RNA (ceRNA) network, namely DLEU1/miR-320b/phosphoribose pyrophosphate synthase 1 (PRPS1) axis. This possible mechanism has been confirmed by bioinformatics and biological experiments. Our findings may provide a new diagnostic biomarker and a potential therapeutic target for CRC.

## Materials and Methods

### Patients and Specimens

Tumor tissues and paired non-tumor tissues were obtained from CRC patients (n=30) by surgery. Patients received no chemotherapy or radiotherapy before surgery. All the participants signed informed consent form. All samples used in this study were approved by the Ethics Committee of Southeast University Affiliated Zhongda Hospital (No. 2017ZDKYSB165).

### qRT-PCR

Total RNAs were obtained from tissues and cells using TRIzol (Applygen, Beijing, China). qRT-PCR was carried by employing Reverse Transcription Kit (Haigene, Harbin, China) for miR-320b and Kit (Haigene, Harbin, China) for DLEU1 and PRPS1. qRT-PCR was performed as previously described (19). The primers were listed in **Table 1**. U6 and β-actin acted as endogenous controls.

**Table 1:** Primer sequences for qRT-PCR.

Primer name	(5’-3’) Primer sequences
miR-320b-Forward	5′-GATGCTGAAAAGCTGGGTTG-3’
miR-320b-Reverse	5′- TATGGTTGTTCTGCTCTCTGTCTC‐3′
U6-Forward	5′- GCTTCGGCAGCACATATACTAAAAT‐3′
U6-Reverse	5′- CGCTTCACGAATTTGCGTGTCAT‐3′
DLEU1-Forward	5′- TGCATTTAAAACCGCCCTGC‐3′
DLEU1-Reverse	5′- TTGAAGAAGGAGACCACGCC‐3′
PRPS1-Forward	5′- GGAAATTGGTGAAAGTGTA‐3′
PRPS1-Reverse	5′-CCACAAGCACCATGCGGTCC‐3′
β-actin-Forward	5′- GACCTGACTGACTACCTCATGAAGAT‐3′
β-actin-Reverse	5′- GTCACACTTCATGATGGAGTTGAAGG‐3′

### Cell Culture and Transfection

CRC cell lines (LoVo, SW620, HCT116 and SW480) and normal cells HIEC were obtained from Cobioer (Nanjing, China) and followed their instructions to culture at 37°C. Sh-LncRNA DLEU1(sequence: CAACGGAAUGUAUCAAUGATT), sh-PRPS1(sequence: GCAGCTCCCACCAGGACTTAT), sh-NC(sequence: TTCTCCGAACGTGTCACGT), miR-320b mimics(sequence: AAAAGCUGGGUUGAGAGGGCAA), NC mimics(sequence: UUCUCCGAACGUGUCACGUTT), miR-320b inhibitors(sequence: UUGCCCUCUCAACCCAGCUUUU) and NC inhibitors(sequence: CAGUACUUUUGUGUAGUACAA) were obtained from RIBOBIO (Guangzhou, China). Cell transfection was conducted following the instruction of Lipofectamine 2000 (Invitrogen, CA, USA). Stably DLEU1-knockdown cell lines were screened out as previously reported (20). In brief, oligonucleotide for small hairpin RNA (shRNA) targeting DLEU1 was synthesized and inserted into the shRNA vector pGPH1/Neo (GenePharma, Shanghai, China). The DLEU1 shRNA vector was transfected into LoVo and SW480 cells with Lipofectamine 3000 (Invitrogen, CA, USA) and selected for 4 weeks with neomycin (1000 μg/ml). Scrambled shRNA (sh-NC) was applied as control. After culturing for 48 h, cells were utilized for follow-up study.

### CCK-8 Assay

Cell viability was assessed using CCK8 kit (MedChemExpress, Shanghai, China). Cells (3000/well) were cultured for 48 h in 96-well plates. Followed by incubating with 10 μL CCK8 for 3-4 h, the absorbance was assessed using GloMax<sp>® System (Promega, WI, USA) at 450 nm (21).

### EdU Assay

The EdU Kit (Beyotime Biotechnology, Nanjing, China) was employed in this study. In brief, after incubation with 10 μM EdU for 2 h, Cells were incubated with 4% formaldehyde, followed by 0.3% Triton X-100 for 10 min. After that, cells were incubated for 30 min with Click Additive Solution in darkness. Finally, 1X Hoechst 33342 was employed for nucleus staining (22).

### Western Blot

Cell lysates were isolated using cell lysis buffer (Cell Signaling Technology, Shanghai, China). The membranes were transferred and blocked at RT for 1 h, followed by primary antibodies incubation: rabbit anti-PCNA (1:1000, ab92552), anti-Ki-67 (1:5000, ab92742), anti-BAX (1:5000, ab32503), anti-Bcl-2 (1:1000, ab59348), anti-Cleaved-caspase3 (1:1000, ab49822), anti-Cleaved-caspase9 (1:1000, ab2324), anti-Cox-2 (1:1000, ab179800), anti-MMP-2 (1:5000, ab92536), anti-MMP-9 (1:10000, ab76003), anti-PRPS1 (1:3000, ab137577) and rabbit anti-β-actin (1:2000, ab8227), and then re-probed with immunoglobulin G (IgG) (1:2000, ab6721) antibody. The chemiluminescence kit (Millipore, Germany) was applied to evaluate the immune response zone and Image J software (ImageJ Software Inc., USA) was used to quantify the integrated density of each band. Antibodies mentioned before were supplied by Abcam (Cambridge, UK).

### Flow Cytometry Assay

The apoptotic rate of CRC cancer cells was evaluated by Annexin V-FITC/PI Apoptosis Detection Kit (Yeasen, Shanghai, China) via flow cytometry. After cultured for 48 h, cells were harvested, following by staining with 10 μL of Annexin V-FITC and PI. Then cells were analyzed by flow cytometer (BD, New Jersey, USA) (23).

### Wound Healing Assay

Cells were cultured in a six-well plate for 24 h. A total of 100 μL pipette tips were used for wounds scratched of each group. Wound was recorded at 0 or 48 h. The distance was assessed by ImageJ software (ImageJ Software Inc., MD, USA). Wound healing rate = (scratch width at 0 h - 48 h)/scratch width at 0 h × 100% (24).

### Transwell Assay

The 24-well transwell chamber (CORNING, New York, USA) was employed for cell invasion following the manufacturer. CRC cells in medium without serum were added to the Matrigel-coated upper chamber. After culturing at 37°C for 24 h, non-invasive cells were eliminated. And invasive cells were counted with Phase-Contrast Microscope (Olympus, Tokyo, Japan). Migration assay was conducted in the same manner with upper chamber without Matrigel (25).

### Subcellular Fractionation

The nuclear and cytoplasm fractions were separated with PARIS Kit (Life Technologies). RNAs in each fraction were extracted. The level of DLEU1 was examined using qRT-PCR. GAPDH and U6 acted as fractionation indicators (26).

### Dual Luciferase Reporter Assay

To confirm that DLEU1 and PRPS1 were targets of miR-320b, LncRNA DLEU1-WT (containing the binding sites of miR-320b at DLEU1), LncRNA DLEU1-Mut (mutation of binding sites), PRPS1-WT (containing the binding sites of miR-320b at PRPS1 3’UTR) and PRPS1-MUT (mutation of binding sites) were cloned into Luciferase Reporter Vector (Invitrogen, CA, USA). LncRNA DLEU1-WT, LncRNA DLEU1-Mut, PFKL-WT or PFKL-MUT were co-transfected with miR-320b mimics into LoVo and SW480 cells. After 48-h transfection, LoVo and SW480 were explored using a Reporter Vector System (Invitrogen, CA, USA) using a Glomax20/20 luminometer (Promega, WI, USA) (27).

### RNA Immunoprecipitation (RIP) Assay

Cells were lysed by RIP lysis buffer (Millipore, USA) and then incubated with magnetic beads conjugated with IgG (1:100, ab109489, Abcam, UK) or Ago2 (1:100, ab32381, Abcam, UK) antibody. Then RNA was purified and investigated using qRT-PCR (28).

### Animal Study

All animal experiments were conducted with the approval of the Institutional Animal Care & Use Committee of China Pharmaceutical University (JN2018035). The nude mice were kept under specific pathogen-free conditions. Water and a basal diet were given ad libitum. Subcutaneously injected 100 μL cell suspension (1 × 107 cells/ml) of stably DLEU1-knockdown or control SW480 cells into the left hind leg of every mouse (n=3 per group). The tumor volume was determined every 5 days using the formula: 0.5 × length × width2. Twenty-five days after the injection, the mice were sacrificed. Tumors were separated and weighted at the same time (28).

### Histology

Tumor tissues were embedded in paraffin, sectioned, dewaxed and rehydrated. Antigens retrieval was conducted using citrate (10 mmol/L, pH 6.0) solution at 100°C for 10 min, followed by immersing the section in 3% hydrogen peroxide for 10 min and incubating overnight with antibody Ki-67 (1:50, ab15580) at 4°C. Incubated the section with IgG (1:2000, ab205718) at room temperature for 2 h. Lastly, 3, 3′-diaminobenzidine tetrahydrochloride was used for visualization, and hematoxylin was used for counter-staining. The nuclei of the tumor cells were calculated by randomly counting 3 fields. The percentage of apoptotic bodies was calculated as: apoptotic/total nuclei × 100 (29).

### Statistical Analysis

Data are shown as mean ± SD. SPSS 21.0 (IBM Corp., NY, USA) was applied for statistical analysis of all data. T-test was used for comparison between two groups, and one-way ANOVA and Tukey’s post-tests were used for multiple groups. The level of significance was set at P < 0.05.

## Results

### DLEU1 Is Highly Expressed in CRC Tissues and Cells

**Figure 1:**
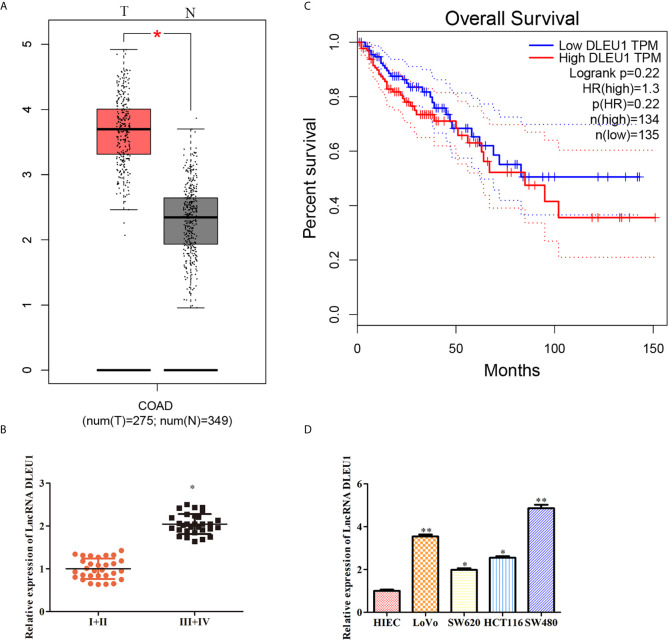
DLEU1 is highly expressed in CRC tissues and cancer cells. **(A)** The expression profile of DLEU1 was obtained from GEPIA (http://gepia.cancer-pku.cn/index.html), *P < 0.05 *vs*. non-tumor tissues. **(B)** The relative expression of DLEU1 was determined by qRT-PCR in CRC tissues of different clinical stage, *P < 0.05 *vs*. stage I + II tissues. **(C)** Overall survival curves showed the association between the DLEU1 level and overall survival of CRC patients were obtained from GEPIA (http://gepia.cancer-pku.cn/index.html). **(D)** The relative expression of DLEU1 was determined by qRT-PCR in CRC cell lines, *P < 0.05, **P < 0.01 *vs*. normal intestinal epithelial cells HIEC.

To evaluate the effect of DLEU1 in CRC, the level of DLEU1 in CRC was obtained from GEPIA. The expression profile exhibited that DLEU1 level was observably increased in CRC tissues compared to non-tumor tissues (**Figure 1A**). In the meantime, the level of DLEU1 in CRC tissues was investigated by qRT-PCR, and our data indicated that DLEU1 level was upregulated with the development of CRC. As shown in **Figure 1B**, DLEU1 level was higher in stage III + IV than I + II. Moreover, the prognosis data demonstrated that the DLEU1 was positively related to the development of CRC patients (**Figure 1C**). Furthermore, the level of DLEU1 in CRC cell lines was investigated by qRT-PCR, which showed that DLEU1 level was higher in CRC cell lines than normal cells HIEC, CRC cell lines LoVo and SW480 were chosen for subsequent experiments (**Figure 1D**).

### Knockdown of DLEU1 Inhibits Proliferation and Stimulates Apoptosis of CRC Cells

**Figure 2:**
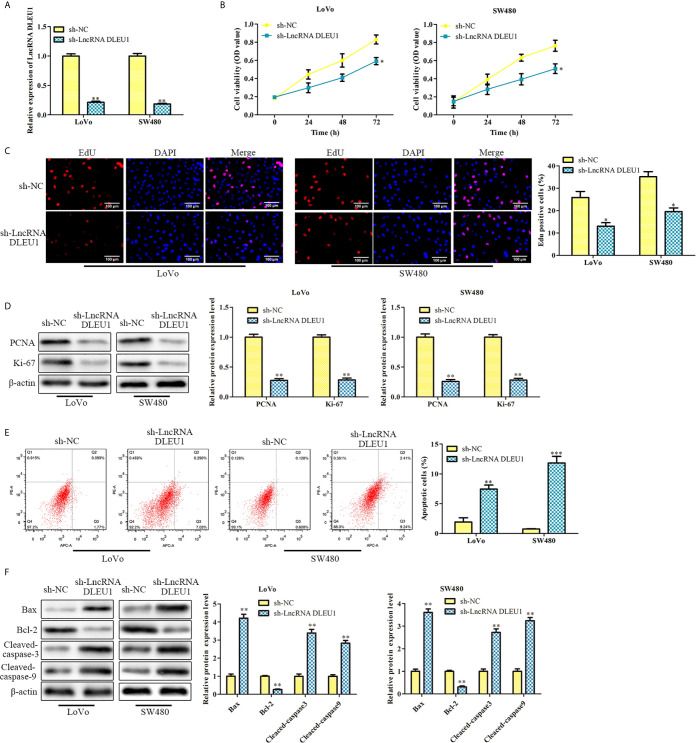
Knockdown of DLEU1 inhibits proliferation and stimulates apoptosis of CRC cells. **(A)** The relative expression of DLEU1 was determined by qRT-PCR in LoVo and SW480 cells. **(B)** The cell viability was determined using CCK-8 assay in LoVo and SW480 cells. **(C)** The cell proliferation was determined using EdU assay in LoVo and SW480. **(D)** The proliferation-related protein levels of PCNA and Ki-67 were investigated by western blot in LoVo and SW480 cells. **(E)** The cell apoptosis was determined using low cytometry assay in LoVo and SW480 cells. **(F)** The apoptosis-related protein levels of Bcl-2, Bax, Cleaved-caspase3 and Cleaved-caspase9 were investigated by western blot in LoVo and SW480 cells. *P < 0.05, **P < 0.01, ***P < 0.001 *vs*. sh-NC.

To further assess the effect of DLEU1 on CRC cell proliferation, sh-LncRNA DLEU1 was used in LoVo and SW480 cells to knock down DLEU1 stably. The qRT-PCR results confirmed that DLEU1 level was observably repressed by sh-LncRNA DLEU1 (**Figure 2A**). We conducted CCK-8 assay and observed that cell viability was diminished by sh-LncRNA DLEU1 (**Figure 2B**). In addition, the results of EdU assay indicated that cell proliferation was diminished by sh-LncRNA DLEU1 (**Figure 2C**). Moreover, proliferation-related protein levels were investigated by western blot. The data demonstrated that PCNA and Ki-67 were markedly suppressed by sh-LncRNA DLEU1 (**Figure 2D**).

In addition, the effect of DLEU1 on CRC cell apoptosis was assessed by flow cytometry. The cell apoptosis was markedly stimulated by sh-LncRNA DLEU1 (**Figure 2E**). Western blot was performed to evaluate the apoptosis-related protein levels, and we observed that Bcl-2 was suppressed, whereas Bax, Cleaved-caspase3 and 9 were remarkably promoted by sh-LncRNA DLEU1 (**Figure 2F**). Taken together, inhibition of DLEU1 repressed CRC cell proliferation and stimulates apoptosis.

### Knockdown of DLEU1 Represses Cell Migration and Invasion

**Figure 3:**
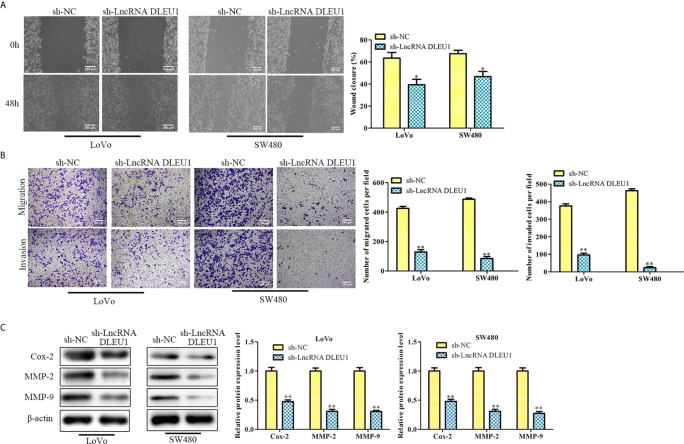
Knockdown of DLEU1 represses cell migration and invasion. **(A)** The cell migration was determined by wound-healing assay in LoVo and SW480 cells. **(B)** The cell invasion was determined using transwell assay in LoVo and SW480 cells. **(C)** The invasion-related protein levels of Cox-2, MMP-2 and MMP-9 were investigated by western blot in LoVo and SW480 cells. *P < 0.05, **P < 0.01 *vs*. sh-NC.

After knockdown of DLEU1 using sh-LncRNA DLEU1 in LoVo and SW480 cells, wound-healing assay was performed to evaluate cell migration. The data indicated that cell migration was markedly suppressed by sh-LncRNA DLEU1 (**Figure 3A**).What’s more, the finding of transwell assay suggested that cell invasion was suppressed by sh-LncRNA DLEU1 (**Figure 3B**). Moreover, invasion-related protein levels were investigated by western blot. The data demonstrated that Cox-2, MMP-2 and MMP-9 were markedly suppressed by sh-LncRNA DLEU1 (**Figure 3C**). Collectively, these results demonstrated that inhibition of DLEU1 repressed cell migration and invasion.

### DLEU1 Interacts With miR-320b in CRC

**Figure 4:**
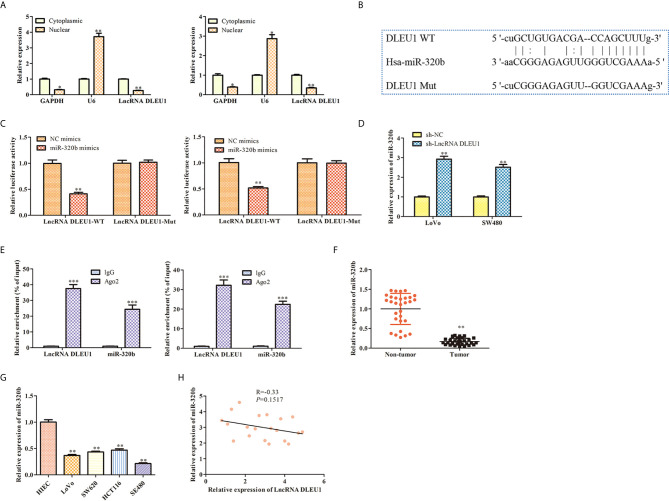
DLEU1 interacts with miR-320b in CRC. **(A)** The levels of DLEU1 in the nuclear and cytoplasm of both LoVo and SW480 cells were determined using qRT-PCR after subcellular fractionation assay, *P < 0.05, **P < 0.01 *vs*. cytoplasmic. **(B)** DLEU1 interacted with miR-320b via bioinformatics analysis. **(C)** DLEU1 interacted with miR-320b using dual luciferase reporter assay, **P < 0.01 *vs*. NC mimic. **(D)** The levels of miR-320b was determined using qRT-PCR, **P < 0.01 *vs*. sh-NC. **(E)** Relative enrichment of DLEU1 and miR-320b in LoVo and SW480 cells was determined using RIP assay, ***P < 0.001 *vs*. IgG. **(F)** The relative expression of miR-320b was determined by qRT-PCR in non-tumor and CRC tissues, **P < 0.01 *vs*. non-tumor tissues. **(G)** The relative expression of miR-320b was determined by qRT-PCR in CRC cell lines, *P < 0.05, **P < 0.01 *vs*. HIEC. **(H)** The correlation between DLEU1 and miR-320b was assessed using Pearson’s correlation analysis.

After subcellular fractionation assay, the levels of DLEU1 in the nuclear and cytoplasm of both LoVo and SW480 cells were determined using qRT-PCR. The data indicated that DLEU1 was mainly in the cytoplasm (**Figure 4A**). Bioinformatics analysis indicated that DLEU1 interacted with miR-320b (**Figure 4B**), which was confirmed by dual luciferase reporter assay. As shown in **Figure 4C**, miR-320b mimics markedly repressed the luciferase activity of LncRNA DLEU1-WT in LoVo and SW480 cells, whereas it had no significant effect on the luciferase activity of LncRNA DLEU1-MUT.We conducted qRT-PCR analysis after DLEU1 knockdown in LoVo and SW480 cells and observed that miR-320b was promoted by sh-LncRNA DLEU1 (**Figure 4D**). Results of RIP assay indicated that both DLEU1 and miR-320b were enriched in Ago2 group (**Figure 4E**), which further demonstrated the interaction between DLEU1 and miR-320b.

Next, the expression of miR-320b in CRC tissues and cell lines was evaluated using qRT-PCR. The finding exhibited that miR-320b level was observably suppressed in CRC tissues and cell lines compared to non-tumor tissues and HIEC (**Figures 4F, G**). Pearson’s correlation analysis results indicated that DLEU1 was negatively correlated with miR-320b in CRC tissues (**Figure 4H**).

### MiR-320b Represses Proliferation and Promotes Apoptosis of CRC Cells

**Figure 5:**
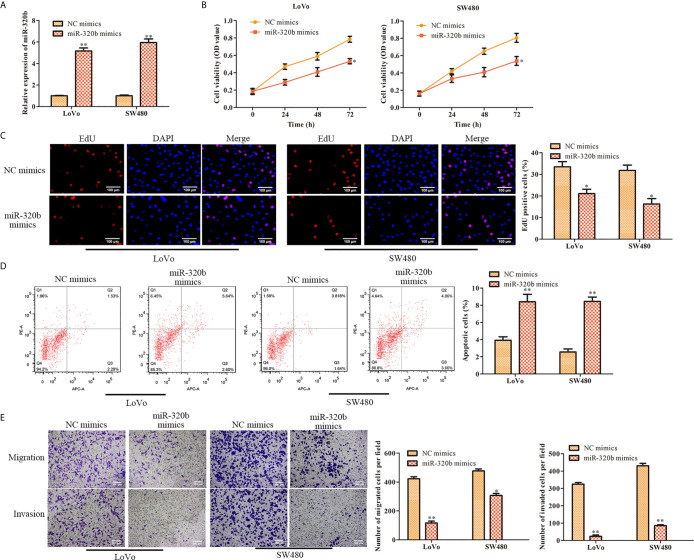
MiR-320b represses proliferation and stimulates apoptosis of CRC cells. **(A)** The relative expression of miR-320b was determined by qRT-PCR in LoVo and SW480 cells. **(B)** The cell viability was determined using CCK-8 assay in LoVo and SW480 cells. **(C)** The cell proliferation was determined using EdU assay in LoVo and SW480. **(D)** The cell apoptosis was determined using flow cytometry assay in LoVo and SW480 cells. **(E)** The cell migration and invasion were determined using transwell assay in LoVo and SW480 cells. *P < 0.05, **P < 0.01 *vs*. NC mimics.

To further assess the effect of miR-320b on CRC cell proliferation, miR-320b mimics was used in LoVo and SW480 cells to upregulate miR-320b. The qRT-PCR results confirmed that miR-320b level was observably elevated by miR-320b mimics (**Figure 5A**). We conducted CCK-8 assay and observed that cell viability was diminished by miR-320b mimics (**Figure 5B**). The results of EdU assay indicated that cell proliferation was suppressed by miR-320b mimics (**Figure 5C**). What’s more, we conducted flow cytometry assay and observed that cell apoptosis was markedly stimulated by miR-320b mimics (**Figure 5D**). Afterwards, we conducted transwell assay and observed that cell migration and invasion were suppressed by miR-320b mimics (**Figure 5E**). Taken together, miR-320b repressed CRC cell proliferation, migration and invasion, while stimulated apoptosis.

### PRPS1 Is a Target Gene of miR-320b

**Figure 6:**
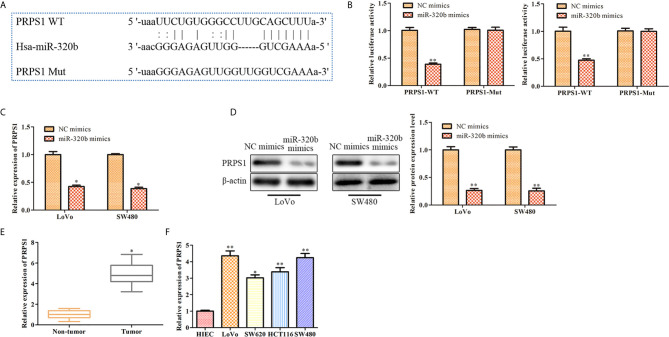
PRPS1 is a target gene of miR-320b. **(A)** PRPS1 was predicted as a target of miR-320b via bioinformatics analysis. **(B)** miR-320b was bound to 3’UTR of PRPS1 using dual luciferase reporter assay, **P < 0.01 *vs*. NC mimics. **(C)** The level of PRPS1 was determined by qRT-PCR, *P < 0.05 *vs*. NC mimics. **(D)** The level of PRPS1 was determined by Western blot, **P < 0.01 *vs*. NC mimics. **(E)** The relative expression of PRPS1 was determined by qRT-PCR in non-tumor and CRC tissues, *P < 0.05 *vs*. non-tumor tissues. **(F)** The relative expression of PRPS1 was determined by qRT-PCR in CRC cell lines, *P < 0.05, **P < 0.01 *vs*. HIEC.

Our bioinformatics analysis result confirmed that PRPS1 was a target gene of miR-320b (**Figure 6A**). Results of dual luciferase reporter assay indicated that miR-320b mimics markedly suppressed the luciferase activity of PRPS1 WT, whereas it had no significant effect when the putative binding sites were mutated (**Figure 6B**). Besides, qRT-PCR and western blot results indicated that both mRNA and protein level of PRPS1 were suppressed by miR-320b mimics. These results verified that PRPS1 was a target gene of miR-320b (**Figures 6C, D**). Next, the expression of PRPS1 in CRC tissues and cell lines was evaluated using qRT-PCR. The finding exhibited that PRPS1 level was observably elevated in CRC tissues and cell lines, compared to non-tumor tissues and HIEC (**Figures 6E, F**).

#### Knockdown of DLEU1 Represses Cell Proliferation, Migration and Invasion While Stimulates Cell Apoptosis *via* miR-320b/PRPS1 Axis

**Figure 7:**
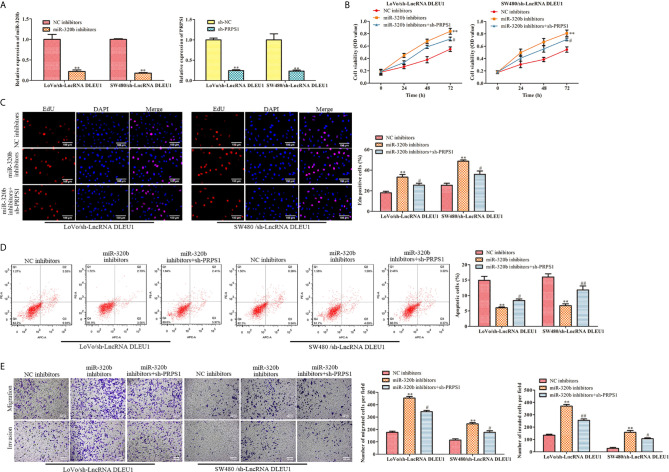
Knockdown of DLEU1 represses cell proliferation, migration and invasion while stimulates cell apoptosis *via* miR-320b/PRPS1 axis. **(A)** The relative expression of miR-320b and PRPS1 were determined by qRT-PCR in LoVo and SW480 cells, **P < 0.01 *vs*. NC inhibitors or sh-NC. **(B)** The cell viability was determined using CCK-8 assay in LoVo and SW480 cells, **P < 0.01 *vs*. NC inhibitors, ^#^ *P* < 0.05 *vs*. miR-320b inhibitors. **(C)** The cell proliferation was determined using EdU assay in LoVo and SW480, **P < 0.01 *vs*. NC inhibitors, ^#^ *P* < 0.05 *vs*. miR-320b inhibitors. **(D)** The cell apoptosis was determined using flow cytometry assay in LoVo and SW480 cells, **P < 0.01 *vs*. NC inhibitors, ^#^ *P* < 0.05, ^##^ *P* < 0.01 *vs*. miR-320b inhibitors. **(E)** The cell migration and invasion were determined using transwell assay in LoVo and SW480 cells, **P < 0.01 *vs*. NC inhibitors, ^#^ *P* < 0.05, ^##^ *P* < 0.01 *vs*. miR-320b inhibitors.

MiR-320b inhibitors was used in LoVo and SW480 cells in which DLEU1 was knocked down stably. qRT-PCR was carried out to detect miR-320b expression and the data exhibited that miR-320b was suppressed by miR-320b inhibitors (**Figure 7A**). In the meantime, sh-PRPS1 was used in LoVo and SW480 cells in which DLEU1 was knocked down stably. The data of qRT-PCR exhibited that PRPS1 was suppressed by sh-PRPS1 (**Figure 7A**).

In order to evaluate the functions of DLEU1/miR-320b/PRPS1 axis, we transfected both miR-320b inhibitors and sh-PRPS1 into LoVo and SW480 cells in which DLEU1 was knocked down stably, followed by CCK-8 and EdU assay. The data of CCK-8 and EdU assay exhibited that cell proliferation was stimulated by miR-320b inhibitors, which was attenuated by sh-PRPS1 (**Figures 7B, C**). The data of flow cytometry assay exhibited that cell apoptosis was suppressed by miR-320b inhibitors, which was reversed by sh-PRPS1 (**Figure 7D**). We conducted transwell assay and observed that cell migration and invasion were stimulated by miR-320b inhibitors, which were attenuated by sh-PRPS1 (**Figure 7E**). Taken together, knockdown of DLEU1 repressed cell proliferation, migration and invasion while stimulated cell apoptosis via miR-320b/PRPS1 axis.

### Inhibition of DLEU1 Suppresses Tumor Growth *In Vivo*

**Figure 8:**
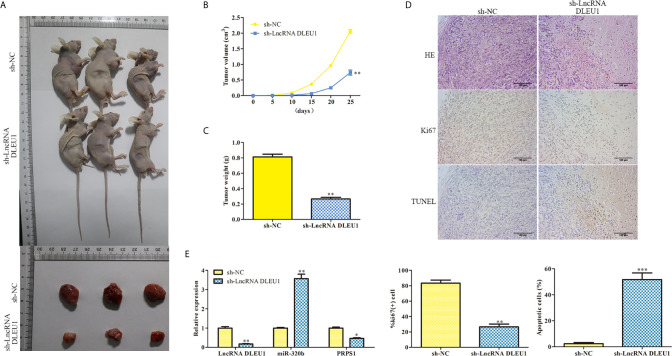
Inhibition of DLEU1 suppresses tumor growth *in vivo.* **(A)** Representative images of xenograft tumors. **(B)** Tumor growth curve. **(C)** Tumor weight. **(D)** Cell necrosis, the Ki-67 expression and cell apoptosis were determined using HE, IHC and TUNEL assay, respectively. **(E)** The expression of DLEU1, miR-320b and PRPS1 were determined by qRT-PCR. *P < 0.05, **P < 0.01, ***P < 0.001 *vs*. sh-NC.

To evaluate the function of DLEU1 *in vivo*, a xenograft model of CRC was established by subcutaneous injection of stably DLEU1-knockdown SW480 cells. As shown in **Figures 8A–C**, DLEU1 inhibition markedly repressed tumour size, growth and weight compared to sh-NC group. HE staining was performed and the results revealed that cell necrosis was significantly elevated by sh-LncRNA DLEU1 (**Figure 8D**). IHC and TUNEL assay were carried out to assess the Ki-67 expression and cell apoptosis in tumor tissues, respectively. Our data revealed that Ki-67 positive cells was alleviated by sh-LncRNA DLEU1, whereas the percentage of apoptotic bodies was elevated by sh-LncRNA DLEU1 (**Figure 8D**). Lastly, the expression of DLEU1, miR-320b and PRPS1 were determined by qRT-PCR. The data revealed that the expression of DLEU1 and PRPS1 were repressed by sh-LncRNA DLEU1, whereas miR-320b was stimulated by sh-LncRNA DLEU1 (**Figure 8E**). The above results confirmed that inhibition of DLEU1 suppressed tumor growth *in vivo*.

## Discussion

Globally, CRC is the third major reason of death caused by cancer (30). Cancer development and metastasis are the major reasons of CRC death, peculiarly for patients in advanced stage (31). Tumor metastasis was often characterized by accelerated invasion and migration. It’s vital to clarify the underlying mechanisms of CRC development.

In recent years, studies have shown that lncRNA DLEU1 is involved in the development of CRC. In the absence of a comprehensive molecular mechanism, no targeted therapeutic drugs have been developed to date. DLEU1 acts as an oncogene in various types of cancers. A study conducted by Zhang et al (32) reveals that DLEU1 plays a tumorigenic role in non-small cell lung cancer. Similarly, DLEU1 is found to stimulate endometrial cancer development (33). Moreover, carcinogenic effect of DLEU1 is found in gastric cancer (34). In this study, our data demonstrated that DLEU1 was highly expressed in CRC tissues and cell lines. We also revealed that knockdown of DLEU1 repressed proliferation, migration and invasion while stimulated apoptosis of CRC cells. These results were consistent with the previous study conducted by Liu et al. (20).

DLEU1 has been reported to interact with miRNAs to regulate gene expression in various cancers. For examples, DLEU1 accelerates the development of pancreatic ductal adenocarcinoma carcinogenesis through the miR-381/CXCR4 axis (35). While in endometrial cancer, DLEU1 regulates SP1 expression *via* sponging miR-490 and aggravates the cancer development (36). In addition, DLEU1 aggravates the progression of ovarian cancer through interacting with miR-490-3p to modulate CDK1 expression (37). To further verify the regulatory mechanism of DLEU1 in CRC, an interaction between DLEU1 and miR-320b was revealed, and that interaction was confirmed by dual luciferase reporter assay and RIP assay in this study.

In plenty of studies, miRNAs, like miR-140 (38), miR-211-5p (39) and miR-29a (40), have been verified to repress proliferation, migration and invasion of tumor cells. As a member of the miR-320 family, miR-320b is found to serve as a tumor suppressor in various cancers (41). A study conducted by Lv et al. (42) reveals that upregulation of miR-320b markedly enhances cell apoptosis and suppresses cell proliferation, migration and invasion in glioma. Besides, through targeting c-Myc, miR-320b attenuates cell proliferation in CRC cells (43). This study exhibited that miR-320b level was observably suppressed in CRC tissues and cell lines. *In vitro* functional studies revealed that miR-320b repressed cell proliferation and stimulated cell apoptosis.

The further mechanism research showed that PRPS1 was a target gene of miR-320b, and its level was observably elevated in CRC tissues and cell lines. PRPS1 is a key enzyme to producing the consensus precursor of nucleotide synthesis (44). Abnormally elevated level of PRPS1 is closely associated with poor prognosis in neuroblastoma, whereas suppression of PRPS1 alleviates cell proliferation and tumor growth (45). Moreover, silence of PRPS1 diminishes cell viability and promotes cell apoptosis in human breast cancer cells (46).

## Conclusion

In summary, our findings elucidated that DLEU1 and PRPS1 were elevated while miR-320b was alleviated in CRC tissues and cell lines. Knockdown of DLEU1 repressed cell proliferation, migration and invasion while stimulated cell apoptosis via miR-320b/PRPS1 axis. Therefore, the DLEU1/miR-320b/PRPS1 axis served as a potential mechanism for CRC development, and our finding provides an exploitable therapeutic target for CRC.

## Data Availability Statement

The raw data supporting the conclusions of this article will be made available by the authors, without undue reservation.

## Ethics Statement

The studies involving human participants were reviewed and approved by The Ethics Committee of Southeast University Affiliated Zhongda Hospital. The patients/participants provided their written informed consent to participate in this study. The animal study was reviewed and approved by China Pharmaceutical University.

## Author Contributions

DX and HY conceived the project and designed the experiments. DX performed the study, analyzed the data, and wrote the paper. YF, WJ, JW, WM, and XD performed the study. DX and YF analyzed the data. HY analyzed the data and critically revised the manuscript. All authors contributed to the article and approved the submitted version.

## Funding 

This study was supported in part by grants from the Health and Family Planning Commission of Jiangsu Province (#H201409, Z2020069), the Health and Family Planning Commission of Nanjing, Jiangsu Province (#YKK16231).

## Conflict of Interest

The authors declare that the research was conducted in the absence of any commercial or financial relationships that could be construed as a potential conflict of interest.
